# Ambient Air Pollution and Apnea and Bradycardia in High-Risk Infants on Home Monitors

**DOI:** 10.1289/ehp.1002739

**Published:** 2011-03-29

**Authors:** Jennifer L. Peel, Mitchel Klein, W. Dana Flanders, James A. Mulholland, Gary Freed, Paige E. Tolbert

**Affiliations:** 1 Department of Environmental and Radiological Health Sciences, Colorado State University, Fort Collins, Colorado, USA; 2 Department of Environmental and Occupational Health, and; 3 Department of Epidemiology, Rollins School of Public Health, Emory University, Atlanta, Georgia, USA; 4 School of Civil and Environmental Engineering, Georgia Institute of Technology, Atlanta, Georgia, USA; 5 Division of Neonatology, School of Medicine, Emory University, Atlanta, Georgia, USA; 6 The Apnea Center, Children’s Healthcare of Atlanta at Egleston, Atlanta, Georgia, USA

**Keywords:** air pollution, apnea, bradycardia, carbon monoxide, infants, nitrogen dioxide, ozone, particulate matter, sulfur dioxide

## Abstract

Background: Evidence suggests that increased ambient air pollution concentrations are associated with health effects, although relatively few studies have specifically examined infants.

Objective: We examined associations of daily ambient air pollution concentrations with central apnea (prolonged pauses in breathing) and bradycardia (low heart rate) events among infants prescribed home cardiorespiratory monitors.

Methods: The home monitors record the electrocardiogram, heart rate, and respiratory effort for detected apnea and bradycardia events in high-risk infants [primarily premature and low birth weight (LBW) infants]. From August 1998 through December 2002, 4,277 infants had 8,960 apnea event-days and 29,450 bradycardia event-days in > 179,000 days of follow-up. We assessed the occurrence of apnea and bradycardia events in relation to speciated particulate matter and gaseous air pollution levels using a 2-day average of air pollution (same day and previous day), adjusting for temporal trends, temperature, and infant age.

Results: We observed associations between bradycardia and 8-hr maximum ozone [odds ratio (OR) = 1.049 per 25-ppb increase; 95% confidence interval (CI), 1.021–1.078] and 1-hr maximum nitrogen dioxide (OR =1.025 per 20-ppb increase; 95% CI, 1.000–1.050). The association with ozone was robust to different methods of control for time trend and specified correlation structure. In secondary analyses, associations of apnea and bradycardia with pollution were generally stronger in infants who were full term and of normal birth weight than in infants who were both premature and LBW.

Conclusions: These results suggest that higher air pollution concentrations may increase the occurrence of apnea and bradycardia in high-risk infants.

Substantial evidence supports an association between ambient air pollution and a wide range of acute respiratory and cardiovascular outcomes, including mortality, hospital admissions, lung function, subclinical markers, and symptom aggravation (American Academy of Pediatrics Committee on Environmental Health 2004; Brook et al. 2010; Pope and Dockery 2006; U.S. Environmental Protection Agency 2006, 2009). The National Research Council has emphasized the need for examination of the groups that are potentially most susceptible to the adverse effects of air pollution, including children and people with underlying cardiovascular and respiratory disease (National Research Council 2004).

Children are considered particularly susceptible to the health effects of air pollution for several reasons, including narrower airways, higher respiratory rates, higher particle deposition rates, greater volume of air breathed per body weight, and increased time spent outdoors compared with older age groups (American Academy of Pediatrics Committee on Environmental Health 2004; Gilliland et al. 1999; Heinrich and Slama 2007; Salvi 2007). Less is known about the activity patterns and air pollution exposure specifically in infants, but infants likely spend less time outdoors than children or even adults. However, infants may be susceptible for additional reasons, including immune, respiratory, and central nervous systems that are not fully developed (American Academy of Pediatrics Committee on Environmental Health 2004; Gilliland et al. 1999; Heinrich and Slama 2007; Salvi 2007; Sly and Pronczuk 2007). Although health outcomes of children have been examined fairly extensively in relation to air pollution, relatively few studies have examined the acute health effects of air pollution in infants, particularly the morbidity effects.

The objective of this study was to examine daily concentrations of ambient air pollution concentrations in relation to apnea (prolonged pauses in breathing) and bradycardia (low heart rate) events detected in infants, using home monitors, from 1 August 1998 through 31 December 2002. During this period, the Aerosol Research and Inhalation Epidemiology Study (ARIES) monitoring site in downtown Atlanta, Georgia, measured a comprehensive suite of pollutants, including particle size fractions and chemical composition as well as gaseous pollutants. The cardiorespiratory events were collected by the Apnea Center of Children’s Healthcare of Atlanta at Egleston, one of the largest referral centers in the United States for home monitoring of infants for apnea or bradycardia. This population of infants on home monitors is considered high risk for cardiorespiratory events and therefore may be particularly susceptible to the adverse effects of air pollution.

## Methods

The study complied with all applicable requirements of the United States; the protocol was approved by the Emory University, Children’s Healthcare of Atlanta, and Colorado State University institutional review boards and was granted a waiver of consent and a Health Insurance Portability and Accountability Act waiver of authorization.

*Air quality data.* Air pollution concentrations were measured at a centrally located monitoring site (the ARIES monitoring site at Jefferson Street) located 4 km northwest of downtown Atlanta, Georgia. Twelve pollutants and metrics were selected *a priori* from the available pollutant measures for use in the primary analyses: 8-hr maximum ozone, 1-hr maximum nitrogen dioxide (NO_2_), 1-hr maximum sulfur dioxide (SO_2_), 1-hr maximum carbon monoxide (CO), 24-hr average total gas-phase oxygenated hydrocarbons (OHC); in addition, 24-hr averages of the following particulate matter (PM) mass measures: PM_10_ (PM with an average aerodynamic diameter < 10 µm), coarse PM (PM with an average aerodynamic diameter between 2.5 and 10 µm), PM_2.5_ (PM with an average aerodynamic diameter < 2.5 µm), PM_2.5_ sulfate, PM_2.5_ elemental carbon (EC), PM_2.5_ organic carbon (OC), and PM_2.5_ water-soluble transition metals. The ARIES pollutant measures have been used in previous health studies (Darrow et al. 2009, 2011; Metzger et al. 2004, 2007; Peel et al. 2005, 2007; Sarnat et al. 2008, 2009; Strickland et al. 2009, 2010; Tolbert et al. 2007); a detailed description of the air quality indices being measured at the ARIES station is available elsewhere (Hansen et al. 2006; Van Loy et al. 2000). Temperature data were collected by the National Climatic Data Center and were measured at Hartsfield-Atlanta International Airport.

*Infant event data.* The patient and event data were obtained from the Apnea Center at Children’s Healthcare of Atlanta at Egleston. Infants and their families are referred by the primary care physician to the Apnea Center for home monitoring; physicians at the center prescribe the home monitor and conduct follow-up and review of the recorded events. Infants are primarily prescribed the home monitor for persistent apnea of prematurity; however, they can also be prescribed for other reasons, including gastroesophageal reflux disease, apparent life-threatening events, and being the sibling of an infant who died of sudden infant death syndrome (SIDS).

Home cardiorespiratory monitors are portable devices that use electrodes placed on the infant’s chest and a belt placed around the infant’s chest to record heart rate, electrocardiogram (ECG), and a measure of chest impedance. The Apnea Center typically recommends that the infants be monitored for about 20 hr/day, especially when the infant is sleeping, in a car seat, or not in the same room as an adult. The monitors use preset parameters to recognize apnea and bradycardia events; an audible alarm sounds when an event is detected. The apnea setting is usually set at 20 sec, as central apneas of this duration or longer are considered clinically significant (American Academy of Pediatrics Committee on Fetus and Newborn 2003; Stokowski 2005). The low heart rate setting is determined largely by the gestational age and chronologic age of the infant; it is usually set at 100 beats/min (bpm) for premature infants < 3 months of age, 80 bpm for term infants < 3 months of age, or 60 bpm for infants ≥ 3 months of age (chronologic age). Impedance is an indirect method of detecting apnea by monitoring the rise and fall of the chest. Therefore, this method detects only central apneas (when there is no respiratory effort) but not obstructive apneas (when there is respiratory effort but not breathing). Bradycardias are often secondary to apneas, occurring when the heart rate drops because the infant is not breathing, although some bradycardias are primarily cardiovascular in nature (not the result of apnea). Therefore, the bradycardia events may include both events that are primarily cardiovascular events as well as bradycardias that are the result of obstructive apnea events.

Information from the home monitors (heart rate, ECG, respiration) is periodically collected by the Apnea Center, generally about once a month or when the center is notified that the infant is experiencing numerous events. Information (all data recorded by the monitor since the previous transmission) is transmitted from the monitor using a modem. Clinicians at the Apnea Center review the event information to confirm that an event is a true event and to determine the type of event (apnea or bradycardia). A false event can occur, for example, if the monitor electrodes fall off of the infant or if the monitor is improperly attached to the infant and is evident in the waveform. If a false event occurs it will be evident in the event information; events determined to be false events were not included in this study. Event information obtained for this study included event date, time, type of event, duration of event, the minimum heart rate for the event (for bradycardia only), the programmed apnea and low heart rate settings, and the compliance recorded for that transmission period (the period since the last transmission; number of days the monitor was used for any portion of the day out of the number of days in the transmission period). Additional information collected from Apnea Center records included date of birth, sex, race, gestational age at birth, birth weight, payment method (Medicaid, private insurance, not insured), the indication for monitor use, and residential ZIP code.

*Statistical analyses.* All analyses were performed using SAS statistical software (version 9.1; SAS Institute Inc., Cary, NC). Apnea and bradycardia events were analyzed separately. Patients who used a home monitor during the study period (1 August 1998–31 December 2002), had a residential ZIP code within the 20-county Atlanta metropolitan statistical area (an area approximately 80 miles by 80 miles), and who were < 6 months of age at the start of their monitor use were included in this study. Follow-up time for each patient was determined by combining all days in the transmission periods during which the patient used the monitor for at least two-thirds of the days (i.e., if, for a particular transmission period, the monitor was used on less than two-thirds of the days in that transmission period, then the entire transmission period was excluded; however, other transmission periods for that patient could still be included in the analysis if compliance was higher for those transmission periods). Follow-up for each subject ended on the last day of an included transmission period, at 6 months of age, or at the end of the study period (31 December 2002).

Descriptive statistics of the air quality data and the outcome data were evaluated. A day of follow-up for an infant was considered an event-day if at least one event of interest (apnea or bradycardia) occurred on that day and a nonevent-day if no events of interest occurred. Because multiple event-days in a subject may be clustered, the primary analysis was a repeated-measures unconditional logistic regression using generalized estimating equations (GEE) (Zeger and Liang 1986). A stationary 45-dependent correlation structure was used in the primary analyses; we chose this correlation structure by examining the correlation of the residuals on each day with residuals from previous days, and it requires that the residual correlation go to zero if the days are > 45 days apart. Events in different subjects were treated as independent. The primary analysis used a 2-day moving average of air pollution concentrations on the same day as the event and one day previous. Individual pollutants were examined in separate models; two-pollutant models were also evaluated. Cubic splines with seasonal knots were used to adjust for long-term temporal trends. Cubic splines with knots at the 25th and 75th percentiles were used to adjust for daily average temperature. The probability of both apnea and bradycardia decreases with increasing age, so a quadratic term for chronologic age was included in the model. Indicator variables for weekend and for federal holidays were also included in the model. Odds ratios (ORs) and 95% confidence intervals (CIs) were calculated for a pollutant increase of approximately 1 SD.

Alternative correlation structures, including autoregressive, exchangeable, and independent correlation structures, were compared with the primary analysis. Models with monthly knots for the cubic splines controlling for time trend were also examined. A sensitivity analysis including only subjects who used the monitor every day in the download time period was performed. We also stratified the subjects by gestational age and birth weight to evaluate heterogeneity of the estimated associations of air pollution and apnea and bradycardia: infants with gestational age < 37 weeks and birth weight < 2,500 g [premature/low birth weight (LBW)] versus infants with gestational age ≥ 37 weeks and birth weight ≥ 2,500 g infants [term/normal birth weight (NBW)]. We used this stratification because most infants in this study were both premature and LBW; because of low numbers, we were not able to look at infants who were both full term and LBW.

## Results

*Air quality data.* Univariate statistics and Spearman rank correlation statistics for the air quality variables are presented in Tables 1 and 2, respectively. Sulfate and OC components made up half of the PM_2.5_ mass (Table 1), with sulfate contributing the largest fraction in summer and OC the largest fraction in winter (data not shown). PM_2.5_ mass and its constituents were moderately to highly correlated (Table 2). As noted in a separate publication (Wade et al. 2006), secondary pollutants (e.g., ozone and sulfate) were generally much more spatially homogeneous and correlated than primary pollutants (e.g., CO, NO_2_, SO_2_, and EC); pollutants of mixed origin (e.g., PM_10_, PM_2.5_, and OC) were intermediate with respect to spatial homogeneity.

**Table 1:** Daily ambient air quality measurements (mean ± SD and 10th and 90th percentiles), 1 August 1998–31 December 2002.

Air quality measure	Percentage of days missing*a*	Mean ± SD	Minimum	10th	Median	90th	Maximum
8-hr ozone (ppb)		11		43.9 ± 24.0		1.25		17.4		39.6		78.0		130.8
1-hr NO_2_ (ppb)		18		41.7 ± 17.9		2.6		21.7		38.7		65.6		109.2
1-hr CO (ppm)		12		1.28 ± 1.11		0.196		0.379		0.865		3.64		7.66
1-hr SO_2_ (ppb)		11		18.3 ± 16.5		0.05		3.46		12.8		40.5		100.8
24-hr OHC (ppb)		26		31.1 ± 15.3		0.7		13.3		29.1		51		91.6
24-hr PM_10_ (µg/m^3^)		9		27.3 ± 12.0		3.85		13.7		25.2		43.8		100.1
24-hr coarse PM (µg/m^3^)		9		9.6 ± 5.4		0.5		3.9		8.7		16.7		50.3
24-hr PM_2.5_ (µg/m^3^)		3		17.8 ± 8.6		1.7		8.2		16.2		29.5		65.8
24-hr PM_2.5_ sulfate (μg/m^3^)		12		5.0 ± 3.4		0.5		1.7		4.1		9.7		20.9
24-hr PM_2.5_ EC (μg/m^3^)		5		1.7 ± 1.2		0.1		0.6		1.4		3.2		11.9
24-hr PM_2.5_ OC (μg/m^3^)		5		4.4 ± 2.4		0.4		2		3.9		7.3		25.9
24-hr PM_2.5_ water-soluble metals (μg/m^3^)		11		0.029 ± 0.024		0.003		0.009		0.022		0.058		0.202
Mean temperature (°C)		0		17.2 ± 7.96		–5.56		6.11		18.3		26.7		32.5
**a**Percentage of days out of the entire study period (1 August 1998–31 December 2002).														

**Table 2:** Spearman correlation coefficients for daily ambient air quality measurements, 1 August 1998–31 December 2002.

Air quality measure	8-hr ozone	1-hr NO_2_	1-hr CO	1-hr SO_2_	24-hr OHC	24-hr PM_10_	24-hr coarse PM	24-hr PM_2.5_	24-hr PM_2.5_ sulfate	24-hr PM_2.5_ EC	24-hr PM_2.5_ OC	24-hr PM_2.5_ water-soluble metals
8-hr ozone		1																						
1-hr NO_2_		0.45		1																				
1-hr CO		0.09		0.59		1																		
1-hr SO_2_		–0.11		0.31		0.39		1																
24-hr OHC		0.40		0.27		0.24		0.04		1														
24-hr PM_10_		0.48		0.50		0.47		0.21		0.49		1												
24-hr coarse PM		0.40		0.39		0.36		0.19		0.39		0.76		1										
24-hr PM_2.5_		0.42		0.47		0.46		0.20		0.46		0.91		0.47		1								
24-hr PM_2.5_ sulfate		0.63		0.36		0.13		0.05		0.32		0.69		0.26		0.78		1						
24-hr PM_2.5_ EC		0.14		0.56		0.70		0.29		0.36		0.66		0.49		0.65		0.29		1				
24-hr PM_2.5_ OC		0.29		0.52		0.61		0.22		0.39		0.71		0.49		0.72		0.38		0.84		1		
24-hr PM_2.5_ water-soluble metals		0.48		0.39		0.36		0.07		0.45		0.71		0.51		0.70		0.74		0.52		0.49		1
Mean temperature (°C)		0.72		0.24		–0.03		–0.24		0.51		0.36		0.26		0.34		0.63		0.06		0.11		0.53


*Infant event data.* A total of 4,277 patients followed by the Apnea Center between 1 August 1998 and 31 December 2002 met the inclusion criteria (Table 3).The mean chronologic age at start of follow-up time in the study was 46 days, and the infants had an average of 42 days included in the study (range 1–134 days). Fifty-three percent of the infants were male. Eighty-four percent of the infants were premature births (< 37 weeks gestation) and 78% were LBW (< 2,500 g). Most infants had private medical insurance (56%) or were covered by Medicaid (41%) (Table 3). Most infants were referred for home monitoring because of previous apnea events (61%) or prematurity (18%); other reasons included gastroesophageal reflux disease (9%), being a sibling of a SIDS baby (3%), previous bradycardia events (2%), apparent life-threatening events (1%), cardiac problems (0.25%), seizures (0.1%), and other (6%).

**Table 3:** Characteristics of infants using monitors between 1 August 1998 and 31 December 2002.

Characteristic	All infants	Infants with at least one recorded apnea event	Infants with at least one recorded bradycardia event
No. of infants		4,277		2,358		3,875
Follow-up time [days (mean ± SD)]		41.9 ± 31.0		47.2 ± 32.7		43.6 ± 31.1
Chronologic age at start of follow-up[days (mean ± SD)]		45.8 ± 35.2		40.6 ± 31.5		46.0 ± 34.4
Male [*n* (%)]		1,766 (53.5)		1,024 (57.0)		1,597 (53.2)
Gestational age < 37 weeks (premature) [*n* (%)]*a*		2,769 (83.8)		1,559 (86.8)		2,586 (86.1)
Birth weight < 2,500 g (LBW) [*n* (%)]*a*		2,494 (78.0)		1,389 (79.8)		2,346 (80.6)
Premature and LBW [*n* (%)]*a*		2,433 (76.3)		1,367 (78.7)		2,289 (78.8)
Full term and NBW [*n* (%)]*a*		459 (14.4)		208 (12.0)		351 (12.1)
Method of payment [*n* (%)]*a*						
Insurance		1,742 (56.0)		971 (57.5)		1,583 (55.9)
Medicaid		1,271 (40.9)		663 (39.3)		1,163 (41.1)
Not insured		95 (3.1)		54 (3.2)		86 (3.0)
Total no. of apnea days in analysis		—		8,960		—
Total no. of bradycardia days in analysis		—		—		29,450
Total no. of follow-up days in analysis		179,207		111,298		168,950
No. of transmissions per patient (mean ± SD)		2.3 ± 1.8		2.8 ± 2.1		2.4 ± 1.9
Compliance per patient (percentage of days used of the total follow-up days within the transmission period) (mean ± SD)		97.6 ± 6.2		97.2 ± 5.3		97.0 ± 5.8
**a**Percentages are based on number of nonmissing subjects.						

Of the eligible infants, 2,358 had at least one apnea event and 3,875 had at least one bradycardia event recorded during the study period. The clinical and demographic characteristics for infants with at least one apnea or at least one bradycardia event were similar to each other and similar to those of the entire population (Table 3). In the 2,358 infants with at least one apnea event recorded, 8,960 apnea event-days (a day on which at least one apnea event was recorded) were recorded in 111,298 total person-days in the analysis. For the 3,875 infants with at least one bradycardia event recorded, 29,450 event-days were recorded in the 168,950 person-days included in the analysis. The monitors were used an average of 97% of days included in the analysis (range, 67–100%, 10th percentile 90%, median 100%) (Table 3). There was no evidence of a strong seasonal or day-of-week trend in the outcome rates (number of daily events/number of infants using the monitor that day). The number of apnea and bradycardia events per the number of subjects monitored each day as well as the number of infants monitored each day was stable over the study period (data not shown).

*Regression results.* ORs and 95% CIs per SD increase in pollution concentration from the primary GEE unconditional logistic regression analyses for apnea and bradycardia using a moving average of pollution lagged 0 and 1 day are presented in Table 4. In our primary analyses, we observed associations between bradycardia and ozone (OR = 1.049 per 25 ppb; 95% CI, 1.021–1.078) and NO_2_ (OR = 1.025 per 20 ppb; 95% CI, 1.000–1.050). In a two-pollutant model, the association with ozone remained elevated, whereas the association with NO_2_ was attenuated (for ozone: OR = 1.041 per 25 ppb; 95% CI, 1.007–1.076; NO_2_: OR = 1.001 per 20 ppb; 95% CI, 0.973–1.031). The OR for ozone and apnea was also elevated, although with a wider CI compared with the more common bradycardia events (OR = 1.036 per 25 ppb; 95% CI, 0.987–1.088).

**Table 4:** Results for GEE unconditional logistic regression analyses examining the association of daily ambient air pollution levels (average of lag 0 and 1) and apnea and bradycardia events in infants on home cardiorespiratory monitors, 1 August 1998–31 December 2002.

Apnea		Bradycardia	
Pollutant	Unit*a*	OR (95% CI)		OR (95% CI)	
8-hr ozone		25 ppb		1.036	(0.987–1.088)		1.049	(1.021–1.078)
1-hr NO_2_		20 ppb		1.011	(0.972–1.052)		1.025	(1.000–1.050)
1-hr CO		1 ppm		0.997	(0.971–1.024)		1.000	(0.984–1.016)
1-hr SO_2_		20 ppb		1.002	(0.964–1.042)		1.002	(0.979–1.025)
24-hr OHC		15 ppb		1.000	(0.958–1.043)		1.010	(0.986–1.035)
24-hr PM_10_		10 μg/m^3^		1.003	(0.977–1.030)		0.995	(0.980–1.011)
24-hr coarse PM		5 μg/m^3^		1.007	(0.977–1.037)		1.005	(0.987–1.023)
24-hr PM_2.5_		10 μg/m^3^		1.002	(0.968–1.037)		0.990	(0.969–1.011)
24-hr PM_2.5_ sulfate		5 μg/m^3^		1.001	(0.945–1.061)		0.991	(0.959–1.025)
24-hr PM_2.5_ EC		1 μg/m^3^		0.999	(0.975–1.023)		0.999	(0.985–1.013)
24-hr PM_2.5_ OC		2 μg/m^3^		1.001	(0.977–1.026)		0.997	(0.982–1.011)
24-hr PM_2.5_ water-soluble metals		0.03 μg/m^3^		0.978	(0.931–1.027)		0.997	(0.970–1.025)
**a**Approximately 1 SD.								

The magnitude of the effect estimates was robust to the choice of correlation structure and to the number of knots in the cubic splines for time (monthly knots versus seasonal knots; data not shown). Results from models including only download periods during which the monitor was used every day yielded results similar to the primary results in Table 4 [see Supplemental Material, Table 1 (http://dx.doi.org/10.1289/ehp.1002739); apnea analysis includes 1,985 subjects, 7,066 apnea days, and 71,560 total days; bradycardia analysis includes 3,299 subjects, 23,276 bradycardia days, and 97,773 total days].

**Figure 1:**
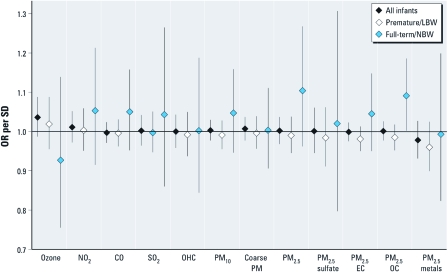
ORs and 95% CIs from analyses examining the association between daily ambient air pollution and apnea events in infants on home monitors, 1 August 1998–31 December 2002; all infants, premature/LBW infants, and full-term/NBW infants.

**Figure 2:**
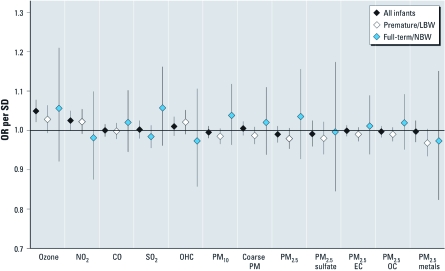
ORs and 95% CIs from analyses examining the association between daily ambient air pollution and bradycardia events in infants on home monitors, 1 August 1998–31 December 2002; all infants, premature/LBW infants, and full-term/NBW infants.

In stratified analyses, the associations of air pollution and both apnea and bradycardia among premature/LBW infants (*n* = 1,367 for apnea; *n* = 2,289 for bradycardia) were generally weaker than the results from the primary analyses and generally consistent with little or no association [Figures 1 and 2; full numerical results are presented in Supplemental Material, Table 2 (http://dx.doi.org/10.1289/ehp.1002739)]. In analyses among full-term/NBW infants (*n* = 208 for apnea and *n* = 351 for bradycardia), there were several strong associations of pollution and apnea and bradycardia events (Figures 1 and 2), although CIs were wide due to the low number of infants. Apnea events in full-term/NBW infants were associated with increased concentrations of the OC component of fine PM (OR = 1.091 per 2 μg/m^3^; 95% CI, 1.002–1.186).

## Discussion

In this study we observed some evidence of acute associations of ambient air pollution with apnea and bradycardia events in infants using home monitors. Associations were generally stronger in infants who were full term and NBW compared with those observed among premature and LBW infants, although the CIs were wide and typically included a null association. Although the immediate and long-term consequences of apnea and bradycardia events in infants are not well defined, these results suggest pollutants could lead to acute respiratory and cardiovascular effects in infants and provide insight regarding the mechanisms by which air pollution can trigger acute health effects.

This is the first study to examine apnea and bradycardia events in infants in relation to ambient air pollution. A growing number of studies have examined the association of ambient air pollution and short-term morbidity in infants. Several studies have reported increases in respiratory hospital admissions among infants in relation to increased ambient pollution concentrations (Barnett et al. 2005; Burnett et al. 1994; Dales et al. 2006; Gouveia and Fletcher 2000a). Several studies have also specifically examined bronchiolitis hospitalizations among infants with mixed results (Karr et al. 2006, 2009a, 2009b; Segala et al. 2008). Three recent studies have reported acute increases in wheeze and other respiratory symptoms in infants in relation to increases in ambient pollution concentrations (Andersen et al. 2008; Pino et al. 2004; Triche et al. 2006). More evidence exists regarding ambient air pollution and infant mortality. Consistent associations between ambient air pollution measures and infant mortality, primarily from respiratory causes and SIDS, have been reported in several countries (Bobak and Leon 1992, 1999; Conceicao et al. 2001; Dales et al. 2004; Ha et al. 2003; Hajat et al. 2007; Kaiser et al. 2004; Knobel et al. 1995; Lipfert et al. 2000; Loomis et al. 1999; Ritz et al. 2006; Saldiva et al. 1994; Woodruff et al. 1997, 2006).

Apneas and bradycardias of short duration are likely common in all infants and are not life threatening (Ramanathan et al. 2001). The consequences of prolonged and more severe events are unclear. Prolonged apnea and bradycardia in the short term could lead to hypoxic-ischemic injury to the infant’s brain; long-term consequences of recurrent events may include neurodevelopmental impairment, although evidence is limited (Stokowski 2005). It is generally accepted that these events are not causally related to SIDS, but there remains much controversy regarding the use of the monitors (Ramanathan et al. 2001). Although their consequences may be unclear, the apnea and bradycardia events in this study are prolonged events determined to be clinically important by clinicians at the Apnea Center.

Hypothesized mechanisms regarding the biologic plausibility for the health effects of inhaled air pollution involve multiple pathways, which include direct irritation of the lungs, pulmonary and systemic inflammation, oxidative stress, vascular alterations, and altered cardiac autonomic function (Brook et al. 2010; Pope and Dockery 2006). Although the underlying cause of apnea and bradycardia events in infants is not clear when there are no obvious precipitating factors, they are thought to be associated with an immature nervous or respiratory system and impaired autonomic control (Martin et al. 2004; Stokowski 2005), making the link with air pollution plausible. Apnea can also be a presenting sign of respiratory infections, including respiratory syncytial virus (Martin et al. 2004; Stokowski 2005). Cigarette smoke exposure has been shown to impair cardiorespiratory control, including altered autonomic nervous control and slower spontaneous recovery of breathing pauses, particularly in preterm infants (Franco et al. 2000; Horne et al. 2004; Schneider et al. 2008). Additionally, Gershan et al. (2004) reported development of apnea and bradycardia in infant rabbits exposed to tobacco glycoprotein.

The stratified analyses provided some evidence that the strongest associations were among the full-term/NBW infants. The weaker results observed among the premature/LBW infants were contrary to what we originally hypothesized. There are several possible explanations for the observed findings. In the premature infants, the immune system may not be fully developed and thus may not able to launch an immune-mediated response to the air pollutants. The premature/LBW infants may also have been more likely to be indoors than full-term infants, potentially decreasing their actual exposure to ambient pollutants. Monitoring of premature infants may also be more prone to motion artifact than with full-term infants; this artifact could lead to misclassification of the outcome (event-days), which would likely be nondifferential with respect to exposure and could therefore lead to a bias toward the null. Finally, given that home monitoring is more common in premature infants, the full-term infants referred for monitoring may represent a high-risk group because of the reasons that they were referred for monitoring. Woodruff et al. (1997) also observed stronger associations between pollution and infant mortality among NBW infants compared with LBW infants; however, several other studies have observed stronger associations among the LBW or premature infants or no difference (Karr et al. 2006; Ritz et al. 2006; Woodruff et al. 2006).

Little is known about the activity patterns and air pollution exposure specifically in infants, but infants likely spend less time outdoors than children or even adults (Jones et al. 2007). Although personal monitoring of air pollution in infants is difficult, Jones et al. (2007) reported that a single ambient monitor provided a reasonable estimate of PM concentrations in infants’ bedrooms. Young infants especially spend most of their time indoors. Given that we observed elevated associations with ozone, which often does not penetrate indoors, there is still some question about whether the ambient ozone concentrations were acting as a surrogate for another correlated pollutant.

This study has several limitations. Although the home monitors record date and time of use, the monitor download summary includes only the number of days in the download period during which the monitor was used (e.g., monitor used 15 of 16 days); the summary does not include information on specific days or times during the day that the monitor was used. Therefore, we likely misclassified event-days as nonevent-days when an infant did not use the monitor on a particular day. This misclassification would result in false-negative values, reducing the sensitivity of the monitor data for identifying event-days. All events detected by the monitors were reviewed by clinicians at the Apnea Center to rule out false events. Therefore, the probability of a false positive is likely very low and the specificity is likely very high. The bias due to nondifferential misclassification of event-days is likely small but will be toward the null if present, and the power of the study could be reduced because of missed events. We attempted to reduce the potential for misclassification of event-days by including only data from monitoring periods during which the monitor was used for at least two-thirds of the days. A secondary analysis was performed including only data from monitoring periods during which the monitor was used every day, and the results were similar to those of the primary analysis [see Supplemental Material, Table 1 (http://dx.doi.org/10.1289/ehp.1002739)].

Additional limitations of this study include the use of a central monitor for pollution measurements, the potential for spurious associations due to the high number of statistical tests performed, and potentially limited generalizability to other locations. Wade et al. (2006) demonstrated that spatial heterogeneity of pollutants in this study varied, with more heterogeneity observed for primary pollutants and much less for secondary pollutants (including ozone). The measurement error resulting from using one centrally located monitor for ambient air pollution concentrations in the entire Atlanta area could potentially attenuate observed associations but is not likely to be responsible for spurious associations. We have attempted to reduce the potential problems associated with multiple testing by using an *a priori* approach for choosing the pollutant metric, pollutant lag structure, and modeling approach; however, we have conducted numerous statistical tests. Finally, these results may not be generalizable to other locations. Behavior such as air conditioning use or time spent outdoors may influence personal exposure levels, which could attenuate the magnitude of the observed associations in comparison with other geographic locations. Factors such as exposure to environmental tobacco smoke are likely not confounders, because they are unlikely to vary day to day with ambient pollution levels; environmental tobacco smoke could be an effect modifier, but we were not able to examine this issue in this study.

## Conclusions

These results suggest that higher air pollution concentrations may increase the occurrence of apnea and bradycardia in high-risk infants. The results also suggest stronger associations in full-term and NBW infants compared with premature and LBW infants. Although it is unclear whether the apnea and bradycardia events have important short-term or long-term consequences for the infants, these results add to the growing evidence demonstrating that infants may be particularly susceptible to health effects of air pollution and could suggest that the effects may be acting through the autonomic nervous pathway given the physiology of apnea and bradycardia events in infants.

## Supplemental Material

(132 KB) PDF
