# Supplementary material for: Ambient Air Pollution and Apnea and Bradycardia in High-Risk Infants on Home Monitors
Source: Environ Health Perspect. 2011 Mar 29;119(9):1321–7. doi: 10.1289/ehp.1002739 (PMC3230388; doi:10.1289/ehp.1002739)
Supplement: (132 KB) PDF [file ehp.1002739.s001.pdf]

Supplemental Material

# Ambient Air Pollution and Apnea and Bradycardia in High-Risk Infants on Home Monitors

Jennifer L. Peel[1], Mitchel Klein[2,3], W. Dana Flanders[3], James A. Mulholland[4], Gary Freed[5,6], Paige E. Tolbert[2,3]

[1] Department of Environmental and Radiological Health Sciences, Colorado State University, Fort Collins, Colorado

[2] Department of Environmental and Occupational Health, Rollins School of Public Health, Emory University, Atlanta, Georgia

[3] Department of Epidemiology, Rollins School of Public Health, Emory University, Atlanta, Georgia

[4] School of Civil and Environmental Engineering, Georgia Institute of Technology

[5] Division of Neonatology, School of Medicine, Emory University, Atlanta, Georgia

[6] The Apnea Center, Children's Healthcare of Atlanta at Egleston, Atlanta, Georgia

Supplemental Material Table 1. Results for GEE unconditional logistic regression analyses examining the association of daily ambient air pollution levels (average of lag 0 and 1) and apnea and bradycardia events in infants on home cardiorespiratory monitors; includes only downloads periods during which the monitor was used 100% of the days, 8/1/1998- 12/31/2002. Apnea analysis includes 1985 subjects, 7066 apnea days, and 71,560 total days; bradycardia analysis includes 3299 subjects, 23,276 bradycardia days, and 97,773 total days.

| | | Apnea | | Bradycardia | |
|---|---|---|---|---|---|
| Pollutant | Unit[a] | OR | 95% CI | OR | 95% CI |
| 8-h ozone | 25 ppb | 1.030 | 0.977, 1.086 | 1.046 | 1.015, 1.078 |
| 1-h nitrogen dioxide | 20 ppb | 1.011 | 0.967, 1.056 | 1.034 | 1.006, 1.062 |
| 1-h carbon monoxide | 1 ppm | 0.999 | 0.970, 1.030 | 1.008 | 0.990, 1.026 |
| 1-h sulfur dioxide | 20 ppb | 0.986 | 0.945, 1.030 | 1.001 | 0.975, 1.011 |
| 24-h oxygenated hydrocarbons | 15 ppb | 1.009 | 0.962, 1.058 | 1.015 | 0.988, 1.044 |
| 24-h $PM_{10}$ | 10 $\mu g/m^3$ | 1.004 | 0.975, 1.033 | 0.993 | 0.975, 1.011 |
| 24-h coarse PM | 5 $\mu g/m^3$ | 1.006 | 0.973, 1.039 | 1.007 | 0.987, 1.027 |
| 24-h $PM_{2.5}$ | 10 $\mu g/m^3$ | 1.006 | 0.968, 1.045 | 0.985 | 0.962, 1.008 |
| 24-h $PM_{2.5}$ sulfate | 5 $\mu g/m^3$ | 0.979 | 0.918, 1.044 | 0.982 | 0.947, 1.019 |
| 24-h $PM_{2.5}$ elemental carbon | 1 $\mu g/m^3$ | 1.008 | 0.981, 1.035 | 1.001 | 0.985, 1.016 |
| 24-h $PM_{2.5}$ organic carbon | 2 $\mu g/m^3$ | 1.005 | 0.978, 1.032 | 0.996 | 0.980, 1.012 |
| 24-h $PM_{2.5}$ water-soluble metals | 0.03 $\mu g/m^3$ | 0.969 | 0.918, 1.022 | 1.002 | 0.971, 1.033 |

Abbreviations: OR, odds ratio; CI, confidence interval; PM, particulate matter
[a] Approximately 1 standard deviation

Supplemental Material Table 2. Results of subgroup analyses examining the association between daily ambient air pollution and apnea and bradycardia events in infants on home monitors, 8/1/1998- 12/31/2002.

| | | Apnea | | Bradycardia | |
|---|---|---|---|---|---|
| Pollutant | Analysis[a] | OR | 95% CI | OR | 95% CI |
| 8-h ozone | Primary analysis | 1.036 | 0.987, 1.088 | 1.049 | 1.021, 1.078 |
| | Premature/LBW | 1.019 | 0.955, 1.088 | 1.028 | 0.993, 1.064 |
| | Full term/NBW | 0.927 | 0.755, 1.139 | 1.056 | 0.921, 1.210 |
| 1-h nitrogen dioxide | Primary analysis | 1.011 | 0.972, 1.052 | 1.025 | 1.000, 1.050 |
| | Premature/LBW | 1.003 | 0.951, 1.059 | 1.022 | 0.991, 1.054 |
| | Full term/NBW | 1.053 | 0.915, 1.213 | 0.981 | 0.875, 1.099 |
| 1-h carbon monoxide | Primary analysis | 0.997 | 0.971, 1.024 | 1.000 | 0.984, 1.016 |
| | Premature/LBW | 0.996 | 0.962, 1.031 | 0.998 | 0.978, 1.019 |
| | Full term/NBW | 1.050 | 0.952, 1.158 | 1.020 | 0.945, 1.102 |
| 1-h sulfur dioxide | Primary analysis | 1.002 | 0.964, 1.042 | 1.002 | 0.979, 1.025 |
| | Premature/LBW | 0.997 | 0.947, 1.051 | 0.984 | 0.955, 1.013 |
| | Full term/NBW | 1.043 | 0.860, 1.265 | 1.057 | 0.961, 1.162 |
| 24-h oxygenated hydrocarbons | Primary analysis | 1.000 | 0.958, 1.043 | 1.010 | 0.986, 1.035 |
| | Premature/LBW | 0.992 | 0.937, 1.050 | 1.021 | 0.990, 1.052 |
| | Full term/NBW | 1.002 | 0.844, 1.188 | 0.973 | 0.857, 1.106 |
| 24-h $PM_{10}$ | Primary analysis | 1.003 | 0.977, 1.030 | 0.995 | 0.980, 1.011 |
| | Premature/LBW | 0.991 | 0.955, 1.028 | 0.985 | 0.965, 1.005 |
| | Full term/NBW | 1.047 | 0.946, 1.159 | 1.038 | 0.963, 1.118 |
| 24-h coarse PM | Primary analysis | 1.007 | 0.977, 1.037 | 1.005 | 0.987, 1.023 |
| | Premature/LBW | 0.996 | 0.956, 1.039 | 0.987 | 0.965, 1.009 |
| | Full term/NBW | 1.003 | 0.906, 1.111 | 1.020 | 0.938, 1.110 |
| 24-h $PM_{2.5}$ | Primary analysis | 1.002 | 0.968, 1.037 | 0.990 | 0.969, 1.011 |
| | Premature/LBW | 0.990 | 0.945, 1.038 | 0.979 | 0.953, 1.006 |
| | Full term/NBW | 1.104 | 0.962, 1.268 | 1.035 | 0.927, 1.156 |
| 24-h $PM_{2.5}$ sulfate | Primary analysis | 1.001 | 0.945, 1.061 | 0.991 | 0.959, 1.025 |
| | Premature/LBW | 0.984 | 0.911, 1.062 | 0.980 | 0.939, 1.022 |
| | Full term/NBW | 1.020 | 0.797, 1.307 | 0.996 | 0.845, 1.174 |
| 24-h $PM_{2.5}$ elemental carbon | Primary analysis | 0.999 | 0.975, 1.023 | 0.999 | 0.985, 1.013 |
| | Premature/LBW | 0.981 | 0.950, 1.013 | 0.990 | 0.972, 1.008 |
| | Full term/NBW | 1.045 | 0.950, 1.148 | 1.011 | 0.939, 1.089 |
| 24-h $PM_{2.5}$ organic carbon | Primary analysis | 1.001 | 0.977, 1.026 | 0.997 | 0.982, 1.011 |
| | Premature/LBW | 0.985 | 0.952, 1.018 | 0.990 | 0.972, 1.008 |
| | Full term/NBW | 1.091 | 1.002, 1.186 | 1.019 | 0.951, 1.092 |
| 24-h $PM_{2.5}$ water-soluble metals | Primary analysis | 0.978 | 0.931, 1.027 | 0.997 | 0.970, 1.025 |
| | Premature/LBW | 0.960 | 0.899, 1.025 | 0.968 | 0.934, 1.004 |
| | Full term/NBW | 0.993 | 0.823, 1.199 | 0.973 | 0.823, 1.151 |

Abbreviations: OR, odds ratio per standard deviation increase; CI, confidence interval; PM, particulate matter

[a]Primary analysis: entire population; Premature/LBW: infants with gestational age < 37 weeks and birth weight <2500 grams ; Full term/NBW: infants with gestational age > =37 weeks and normal birth weight >=2500 grams.
